# Reproducibility and Diagnostic Utility of a Simplified Oil Red O Test in Infant Bronchoalveolar Lavage Samples

**DOI:** 10.1002/dc.70031

**Published:** 2025-10-12

**Authors:** Emily Wheeler, Jennifer Kernodle‐Zimmer, Melissa Randolph, Harvey Cramer, Hector Mesa

**Affiliations:** ^1^ Division of Cytopathology, Department of Pathology and Laboratory Medicine Indiana University School of Medicine Indianapolis Indiana USA; ^2^ University of Maryland School of Medicine Baltimore MD USA

**Keywords:** alveolar macrophages, bronchoalveolar lavage fluid/cytological techniques, diagnostic techniques and procedures, newborn, oil red O, pulmonary aspiration/infant

## Abstract

**Introduction:**

Aspiration in infants is a diagnostic challenge. The lipid‐laden macrophage index (LLMI) developed in 1987 has been used as a supportive test; however, numerous recent studies have questioned its value and reproducibility. We evaluated a simplified LLMI in bronchoalveolar lavage (BAL) specimens from a pediatric cohort to assess its diagnostic utility.

**Methods:**

BALs from infants were prospectively collected over a 6‐month period for Oil Red O (ORO) staining to evaluate aspiration. BALs from adults with non‐aspiration pathologies were simultaneously collected for comparison. Clinical and demographic data were gathered to assess the diagnostic accuracy of the test. Only samples containing ≥ 100 evaluable macrophages and free of obscuring blood or inflammation were included. Positive staining was assessed at low magnification (10×), with only clearly positive cells (Colombo‐Hallberg scores 3 and 4) considered. A dichotomous threshold of < 50% or ≥ 50% positive macrophages was established through multidisciplinary consensus. To ensure consistency, a training session was conducted for the entire cytopathology division on the newly developed interpretation criteria.

**Results:**

88/134 (66%) pediatric BAL samples with suspected aspiration and 63/75 (84%) adult samples with various non‐aspiration pathologies were adequate for analysis. Aspiration status in children was determined using multidisciplinary aerodigestive group evaluation (MAGE) and videofluoroscopic swallow study (VFSS). Test performance was assessed at various cutoffs. In the pediatric cohort (mean age 16.5 months, 58% male), aspiration was diagnosed in 47% by MAGE. Strong associations were seen with atopia/asthma (83%), functional dysphagia (64%), and congenital/developmental disorders (43%). A significant difference in ≥ 50% lipid‐laden macrophage involvement was observed between pediatric (12%) and adult (51%) samples (*p* < 0.00001). Using MAGE and VFSS as gold standards, the test showed poor discriminatory power for detecting aspiration in infants (AUC 0.506–0.587). A 10% cutoff yielded the best performance (AUC 0.587, sensitivity 27%, specificity 93%), while a 50% cutoff offered practical advantages in workflow and reproducibility.

**Conclusions:**

The modified LLMI demonstrates limited diagnostic value for aspiration in infants. While a 10% cutoff offers slightly improved performance, the test may be phased out in favor of more reliable diagnostic methods.

## 1 Introduction

The use of Oil Red O (ORO) staining in bronchoalveolar lavage (BAL) procedures has been on the rise, largely due to updated protocols from pediatric multidisciplinary aerodigestive teams aimed at evaluating children with suspected aspiration. Given that maternal milk and infant formula typically contain about 3%–5% lipids [1] Colombo and Hallberg [2] introduced the lipid‐laden macrophage index (LLMI) in 1987. This index was designed as a risk scoring system to quantify lipid accumulation in alveolar macrophages. It involves examining 100 macrophages and assigning each a score from 0 to 4, depending on the percentage of cytoplasm filled with ORO‐positive lipid vacuoles: 0 for none, 1 for less than 25%, 2 for 25%–50%, 3 for 50%–75%, and 4 for more than 75%. A total score above 90 was considered indicative of a high risk of aspiration.

However, studies published since the early 2000s have raised concerns about the LLMI. Cytologists/cytopathologists have found it to be a complex and resource‐intensive method, with poor reproducibility both within and between observers [3, 4, 5, 6, 7, 8]. When we attempted to implement the LLMI in our own laboratory, we encountered the same challenges. These findings led us to reach out to our multidisciplinary aerodigestive team to discuss the possibility of developing a reporting system that would be simpler, more reproducible, less labor‐intensive, and also clinically useful for evaluating patients with suspected aspiration.

## 2 Materials and Methods

Our laboratory optimized the ORO staining protocol to reduce non‐specific background staining, which was highly prevalent in our samples. The final procedure is described in Table 1.

**TABLE 1:** Oil Red O stain procedure.

Step	Procedure
1	Fix slides in Baker's formal calcium or 10% formalin for 10 min
2	Wash slides in distilled water
3	Stain in filtered Oil Red O solution for 15 min at room temperature
4	Rinse slides in running tap water for 20 min
5	Rinse slides in distilled water
6	Stain in hematoxylin for 30 s
7	Rinse in distilled water
8	Mount slides with aqueous mounting media (do not press to avoid fat damage)

BALs from infants, requested for ORO staining by our institutional multidisciplinary aerodigestive group over a 6‐month period, were prospectively collected. Demographic and clinical information, including age, gender, pre‐test diagnosis, and post‐test assessment by the multidisciplinary aerodigestive group evaluation (MAGE), were also collected to determine the diagnostic accuracy of the test for detecting aspiration. Cases with incomplete diagnostic assessment or deemed inadequate for cytologic evaluation were excluded from the study.

**FIGURE 1:**
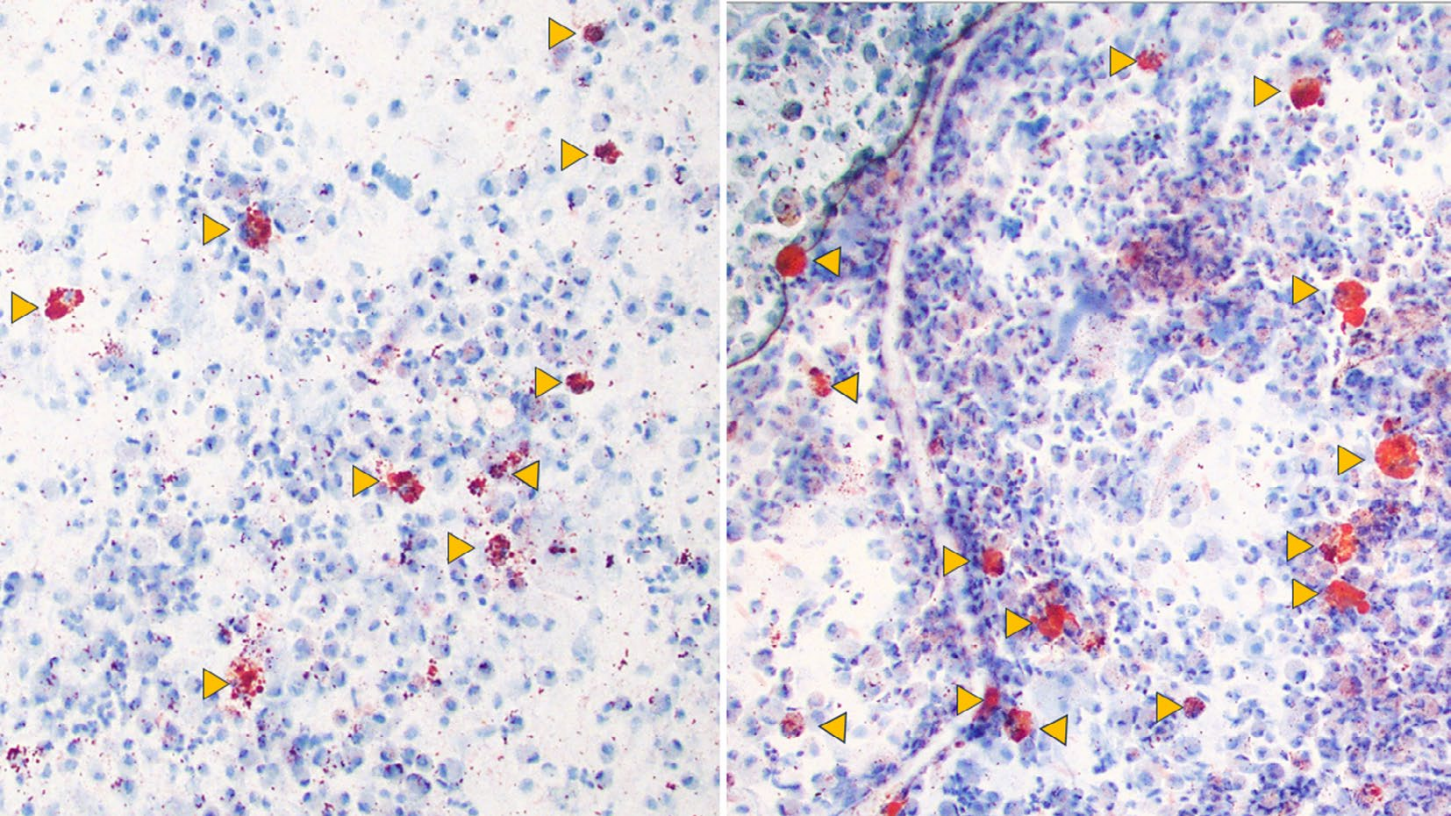
Oil Red O (ORO) stain of bronchoalveolar lavage (BAL) in 2 separate samples at 10× magnification. For scoring purposes, only macrophages that are clearly positive at low magnification (arrowheads) are considered positive. For clinical interpretation, a qualitative threshold of < 50% or > 50% positive macrophages was established in collaboration with the multidisciplinary clinical team. For investigational analysis, discrete scoring was performed by counting positive macrophages first, followed by negative ones, until a total of 100 cells were evaluated. This score was correlated with the outcome “aspiration” to determine the optimal diagnostic cutoff. [Color figure can be viewed at wileyonlinelibrary.com]

Adequacy was defined as having ≥ 100 evaluable macrophages and the absence of obscuring blood or inflammation. Percentage estimation was performed only at low magnification (10×) without formal counting. Only clearly positive macrophages at low magnification (scores 3 and 4 on the Colombo‐Hallberg scale) were considered (Figure 1), and the evaluation was performed in fields with the highest number of positive cells.

A dichotomous scale (< 50% or ≥ 50% positive macrophages) was proposed as an empiric, clinically relevant cutoff after discussion among our clinical multidisciplinary group, which included pediatric gastroenterologists, pulmonologists, speech therapists, and the cytopathology division.

A training session for the entire cytopathology division was conducted to implement the newly developed interpretation criteria. Feedback about the feasibility of this new assessment was obtained at the monthly departmental meeting.

Adult samples were included for comparison and to determine the frequency of ORO‐positive macrophages in individuals without a risk for aspiration.

To evaluate the reproducibility of the method, a correlation analysis was performed on 55 samples assessed independently by two cytotechnologists (EW and MR).

In the pediatric cohort, a second evaluation was conducted by two authors (EW and HM) using discrete scores by counting 100 cells at 10× magnification to determine if a better cutoff value existed. Similar to the original evaluation, only ORO (+) macrophages with scores of 3 and 4 on the Colombo‐Hallberg scale were counted using a cell counter. ORO (+) macrophages were counted first to establish the score in cases with more than 100 cells per field. In cases with fewer than 100 macrophages per 10× field, several fields were scored until 100 cells were counted, always counting the ORO (+) macrophages first (Figure 1). Although the score does not strictly represent a percentage, it was expressed as such for reporting and analytic purposes. Aspiration status was determined through multidisciplinary group evaluation and retrieved from the electronic medical records.

The statistical software SPSS (IBM SPSS Statistics for Windows, Version 27.0, IBM Corp., 2020) was used to calculate the test characteristics and plot the Receiver Operating Curve (ROC) to evaluate the test's performance using “aspiration” as the binary outcome. Calculations were performed using the adjudication established through the MAGE and the results of the Videofluoroscopic Swallow Study test as gold standards.

## 3 Results

The results of the correlation analysis are depicted in Table 2.

**TABLE 2:** Pearson correlation analysis for 2 observers, *N* = 55.

Metric	Value
Pearson correlation coefficient	0.81
*p*	< 0.0001
Agreement rate	92.3%

The Pearson correlation coefficient of 0.81 demonstrated a strong, highly significant correlation between the two observers, supporting the reproducibility of the method.

Out of 134 pediatric samples, 88 (66%) were adequate for evaluation. Clinical indications included aspiration (40%), chronic cough (16%), dysphagia (2%), abnormal breathing (6%), combinations of the afore mentioned symptoms (35%), and others (5%) (Table 3). The cohort consisted of 50 males (58%) and 37 females (42%), for a male‐to‐female ratio of 1.4:1. The mean age was 16.5 ± 10 months (range 1–63 months).

**TABLE 3:** Reason for ORO BAL test and post‐evaluation diagnoses by MAGE.

Reason for ORO BAL request	*n*	%	Postevaluation diagnosis^a^	*n*	%
Aspiration	35	40	Aspiration	41	47
Cough	14	16	No aspiration	47	53
Cough and aspiration	16	18	Congenital/developmental disorder	56	64
Cough and dysphagia/difficulty feeding	10	11	Atopia/asthma	23	26
Respiratory symptoms	5	6	Functional dysphagia	19	22
Dysphagia/difficulty feeding	2	2	Infection	21	24
Aspiration and respiratory symptoms	1	1			
Aerodigestive malformation	1	1			
AED malformation and aspiration	2	2			
Pneumonia	1	1			
Hemoptysis	1	1			

Abbreviation: MAGE, multidisciplinary group evaluation.

^a^ Postevaluation diagnoses were not mutually exclusive and included various comorbid conditions.

The MAGE of the cohort concluded that 47% of the infants had evidence of aspiration. The most common underlying conditions present in this cohort included congenital/developmental abnormalities (64%), atopia/asthma (22%), functional dysphagia (22%), and infection (24%).

Stratifying underlying conditions by aspiration status, as determined by MAGE, revealed variable associations with aspiration: strongest for atopia/asthma: 83%, followed by functional dysphagia: 64%, congenital/developmental disorders: 43%, and their combinations. No association was observed with infection (Table 4).

**TABLE 4:** Comorbid conditions by aspiration status assigned by MAGE.

Disease/condition	Aspiration (*n*)	No aspiration (*n*)	% aspiration
Congenital/developmental disorder (C&D‐Dis.)	15	20	43
Functional dysphagia	7	4	64
Atopia/asthma	5	1	83
C&D‐Dis. and atopia/asthma	5	3	63
C&D‐Dis. and functional dysphagia	3	0	100
C&D‐Dis. and infection	2	6	25
Atopia/asthma and functional dysphagia	2	1	67
C&D‐Dis. and infection and functional dysphagia	1	0	100
Atopia/asthma and infection	1	4	20
Infection	0	7	0
C&D‐Dis and atopia/asthma and functional dysphagia	0	1	0

Abbreviation: MAGE, multidisciplinary group evaluation.

In this cohort, cases with less than 50% involvement comprised 77 (88%), while those with 50% or more involvement were 11 (12%).

For the adult samples, 63 out of 75 (84%) were evaluable. Clinical indications included pneumonia/opacities/infiltrates (28 cases, 44%), cancer (19 cases, 30%), mass/nodule (15 cases, 23%), adenopathy (7 cases, 11%), and others (less than 5%). The cohort consisted of 36 males and 27 females for a male‐to‐female ratio of 1.3:1. Mean age was 61.5 ± 17 years (range 10–83). Cases with less than 50% involvement comprised 31 (48%), while those with 50% or more involvement were 32 (51%).

A Chi‐square test showed a highly significant difference in the ≥ 50% threshold between pediatric and adult samples (*p* < 0.00001).

Table 5 depicts the test performance characteristics of the new method in the pediatric population at different cutoff values, with aspiration as the binary outcome. Separate calculations were performed using either MAGE (*n* = 88) or the results of the Videofluoroscopic Swallow Study (VFSS) (*n* = 41) as gold standards. The prevalence of aspiration was high with both methods: 47% with MAGE and 65% with VFSS.

**TABLE 5:** Performance characteristics of ORO‐BAL test at different cutoff values.

Cutoff	Gold Std.	Sens%	Spec%	PPV%	NPV%
1	MAGE	97.6	6.4	47.6	75
	VFSS	96.2	7.1	65.8	50
5	MAGE	48.8	48.9	45.5	52.3
	VFSS	46.2	64.3	70.6	39.1
10	MAGE	29.3	74.5	50	54.7
	VFSS	26.9	92.9	87.5	40.6
20	MAGE	21.9	87.2	60	56.2
	VFSS	15.4	92.9	80	37.1
30	MAGE	22	91.5	69.2	57.3
	VFSS	15.4	92.9	80	37.1
50	MAGE	19.5	93.6	72.7	57.1
	VFSS	15.4	92.9	80	37.1

Abbreviations: MAGE, multidisciplinary aerodigestive group evaluation; VFSS, videofluoroscopic swallow study.

**FIGURE 2:**
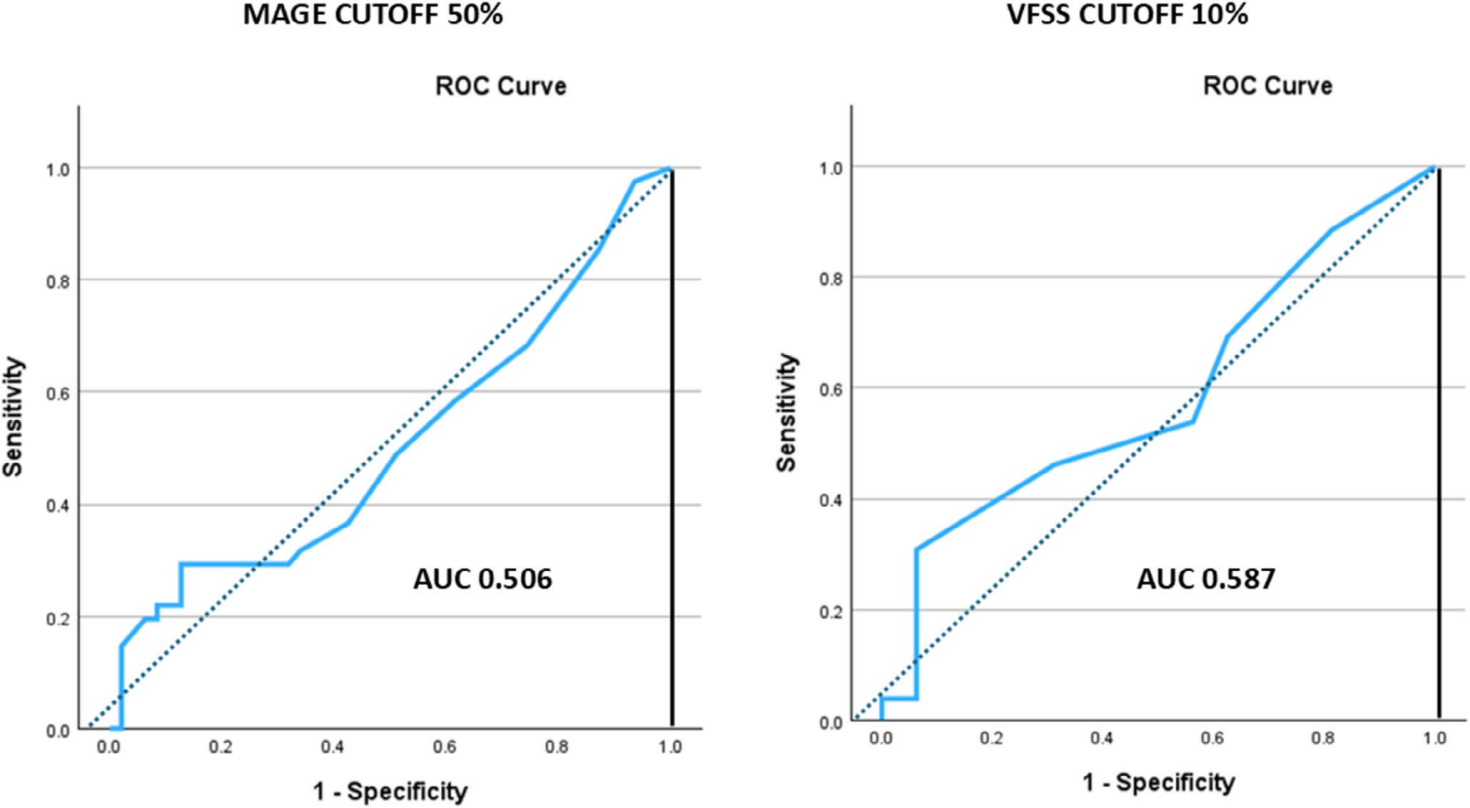
Receiver operating characteristic (ROC) plots for the ORO stain macrophage index in bronchoalveolar lavage (BAL). Left: ROC curve using an empiric cutoff of 50%, with MAGE as the reference standard. Right: ROC curve using an optimized cutoff of 10%, with VFSS as the reference standard. The 10% cutoff with VFSS demonstrated the best overall performance among all tested thresholds and reference standards. However, both models exhibited poor predictive accuracy and limited discriminative ability for detecting aspiration. [Color figure can be viewed at wileyonlinelibrary.com]

Using MAGE as gold standard, the test showed poor discriminatory power for detecting aspiration, with optimal results at a cutoff of 30%, and little variation in test performance above this value. The arbitrary empiric cutoff of 50% had an area under the curve (AUC) of 0.506 on the ROC plot, sensitivity (sens) of 19.5%, specificity (spec) of 93.6%, positive predictive value (PPV) 72.7% and negative predictive value (NPV) of 57% (Figure 2, Table 4).

Using VFSS as the gold standard, optimal results were obtained with a cutoff value of 10%, with little variation in test performance above this value. Although slightly better performance was observed with this gold standard, the test still showed poor discriminatory power to detect aspiration with an AUC of 0.587, sens of 27%, spec of 93%, PPV of 88%, NPV of 41% (Figure 2, Table 4).

## 4 Discussion

Recurrent aspiration in infants can lead to serious complications such as chronic lung disease, recurrent pneumonia, and growth retardation due to feeding difficulties [9]. Early identification allows for timely interventions that may improve respiratory and nutritional outcomes. However, diagnosis is often challenging due to the limited communicative abilities of infants, frequent associations with congenital and developmental disorders, subtlety of symptoms, variability in presentation, and limitations of current diagnostic tools. Instrumental assessments like videofluoroscopic swallow studies provide the most direct objective evidence of aspiration but may not capture intermittent aspiration. A definitive diagnosis of aspiration is often complex and typically requires a collaborative evaluation of clinical findings, diagnostic procedures, and imaging by a multidisciplinary team, including otolaryngologists, pulmonologists, gastroenterologists, and speech‐language pathologists [9].

The LLMI, assessed using Oil Red O staining in bronchoalveolar lavage fluid, was first introduced by Colombo and Hallberg in 1987 [2]. It was proposed as a supplementary test to evaluate the risk of aspiration that could be incorporated into the standard panel of cytological analyses routinely performed on BAL samples. The limitations and disadvantages of this test have been reported in a number of previous studies [4, 5, 6, 7]. A major challenge in interpreting the LLMI lies in the variability of the Oil Red O (ORO) stain quality and the limited reproducibility of its interpretation, as previously reported by Torous et al. [8]. Additionally, the LLMI is highly labor‐intensive, as it requires the complex task of summing a 0–4 score across 100 macrophages to enable risk stratification. In the initial phase of our study, we focused on optimizing the Oil Red O (ORO) staining protocol for bronchoalveolar lavage (BAL) samples, as outlined in Table 1. Once consistent staining results were achieved, we developed a simplified, less labor‐intensive, and more reproducible method for interpreting ORO‐positive macrophages. This new approach was collaboratively designed by our cytology team and multidisciplinary aerodigestive group, and it employed an empirically derived binary threshold of 50% lipid‐laden macrophages to guide clinical decision‐making. The cytology team found the revised scoring system to be practical, easy to implement, and time‐efficient, while the clinical team reported it as a valuable tool for evaluating suspected aspiration cases. Correlation analysis between the two cytotechnologists involved in method validation yielded a Pearson correlation coefficient of 0.81 (*p* < 0.0001), indicating high inter‐observer reproducibility.

Evaluation of this new method in pediatric patients with suspected aspiration and a control adult cohort with various non‐aspiration‐related disorders revealed significant differences between the two groups. Among pediatric samples, the majority (88%) had less than 50% lipid‐laden macrophages. In contrast, 51% of adult samples were ≥ 50% threshold. This difference was highly significant (*p* < 0.00001), suggesting that the test had greater specificity for diagnosing aspiration in pediatric populations and lacked utility in adults since aspiration was not a diagnostic consideration in this cohort.

In the second phase of our study, we aimed to refine the diagnostic threshold for aspiration in pediatric patients using the simplified method. This was done by correlating various cutoff values with aspiration status, as determined by multidisciplinary aerodigestive team evaluation and videofluoroscopic swallow studies. The analysis showed that the empirically selected 50% cutoff had poor diagnostic performance, with an area under the curve (AUC) of 0.506, indicating a very limited ability to distinguish between aspirators and non‐aspirators. The actual performance may be even lower, as the 50% threshold was used by the multidisciplinary team during adjudication, which very likely introduced confirmation bias. The optimal cutoff value was 30%, with a Youden's Index of 112.5, a sensitivity of 22%, specificity of 92%, PPV 69%, and NPV 57%; however, this threshold also performed poorly as a diagnostic test and is also likely affected by confirmation bias for the same reason described above.

The optimal cutoff using VFSS was 10%, demonstrating slightly better performance with an AUC of 0.587, a sensitivity of 27%, specificity of 93%, PPV of 88%, and NPV of 41%. This cutoff is likely more reliable, as VFSS is a more robust diagnostic standard in terms of reproducibility, reliability, and accuracy, and its interpretation was not influenced by the ORO staining results in BAL samples. The clinical utility of this test is further diminished when considering the high prevalence of aspiration in our patient cohort: 47% by MAGE and 65% by VFSS. In such a population, a test with high sensitivity would be more valuable for screening purposes. However, the LLMI test, including our modification, primarily offers high specificity, limiting its usefulness for detecting aspiration.

In infants, our modified LLMI showed low sensitivity and relatively high specificity, resulting in more false negatives than false positives. In adults, the test lacked both sensitivity and specificity, as none had clinical suspicion of aspiration. The high prevalence of aspiration in our cohort likely reflects referral bias, given the study was conducted at a large quaternary care center. These factors may limit the generalizability of our findings.

Although lipid laden macrophages (LLM) are commonly associated with aspiration, they are not specific to this condition. Conditions associated with increased LLM in BAL in pediatric patients include bronchiectasis, particularly in children with cystic fibrosis, acute lung injury, pulmonary infections, airway obstruction, endogenous or exogenous lipoid pneumonia, and parenteral nutrition, which may result in foamy macrophages due to impaired clearance or increased levels of intravascular or intra‐alveolar lipids [3, 5, 10]. In adults, they have been identified in multiple diseases including COPD, asthma, idiopathic pulmonary fibrosis, and infections [10].

Another limitation of oil‐red‐O staining is variability in technique across laboratories, which affects both LLMI and our modified scoring system. The cutoffs used in our approach are based on our institution's staining protocol, as described in the Methods section, and may not be applicable to other protocols without further validation.

## 5 Conclusions

We describe a modified version of the Colombo‐Hallberg LLMI for use in BAL specimens, developed to improve reproducibility, reduce labor demands, and provide clinical utility. Consistent with previous studies, we found that the test has limited diagnostic value for detecting aspiration in infants, regardless of the cutoff applied, and likely should be phased out. However, given that multidisciplinary teams continue to request the test, and its use has increased, a 50% cutoff offers practical advantages, including ease of implementation, shorter evaluation time, and good reproducibility. A 10% cutoff demonstrated the best performance for predicting aspiration and can also be implemented effectively using the methodology described in this article or adapted to protocols validated by individual laboratories.

## Conflicts of Interest

The authors declare no conflicts of interest.

## Data Availability

The data of this study are available on request from the corresponding author. The data are not publicly available due to privacy restrictions.
